# An Anthracene-Based Tripodal Chemosensor for Anion Sensing

**DOI:** 10.3390/ijerph7052057

**Published:** 2010-05-04

**Authors:** Whitney A. Quinn, Musabbir A. Saeed, Douglas R. Powell, Md. Alamgir Hossain

**Affiliations:** 1 Department of Chemistry and Biochemistry, Jackson State University, Jackson, MS 39217, USA; E-Mails: sunshinequinn@yahoo.com (W.A.Q.); m.a.saeed@gmail.com (M.A.S.); 2 Department of Chemistry and Biochemistry, University of Oklahoma, Norman, OK 73019, USA; E-Mail: d-powell@ou.edu

**Keywords:** Tren, chemosensor, anion complex, fluorescence titrations, anion receptor

## Abstract

An anthracene-based tripodal ligand was synthesized from the condensation of tren with 9-anthraldehyde, and the subsequent reduction with sodium borohydride. The neutral ligand was protonated from the reaction with *p*-toluenesulfonic acid to give a triply charged chemosensor that was examined for its anion binding ability toward fluoride, chloride, bromide, sulfate and nitrate by the fluorescence spectroscopy in DMSO. The addition of an anion to the ligand resulted in an enhancement in fluorescence intensity at the excitation of 310 nm. Analysis of the spectral changes suggested that the ligand formed a 1:1 complex with each of the anions, showing strong affinity for fluoride and sulfate in DMSO. The unsubstituted tren was reacted with sulfuric acid to form a sulfate complex and the structure was determined by the X-ray crystallography. Analysis of the complex revealed that three sulfates are held between two ligands by multiple hydrogen bonding interactions with protonated amines.

## 1. Introduction

Selective binding of anions occurs in many physiological and enzymatic processes in life [1]. It is known that the majority of enzyme substrates and cofactors are anions, often in the form of phosphate residues such as adenosine triphosphate, adenosine diphosphate or even simple phosphate [2]. Anions such as sulfate and chloride are important species in many biochemical processes [3]. There are several anionic species which are directly related to adverse health effects in humans. For example, exposure to high doses of nitrate and nitrite are related with an adverse health effect in human such as cancer [4], birth defects [5], cardiovascular problems [6] and thyroid hypertrophy [7]. Anion binding to proteins (e.g., sulfate and phosphate binding to proteins) is important in many physiological and metabolic processes [8]. Nitrate, sulfate, and halides are the common contaminants in soil and water, and have negative effects on human health [9]. Thus, there is a critical need to develop new systems that are suitable for sensing and binding anions with environmental and biomedical relevance.

**Scheme 1.**
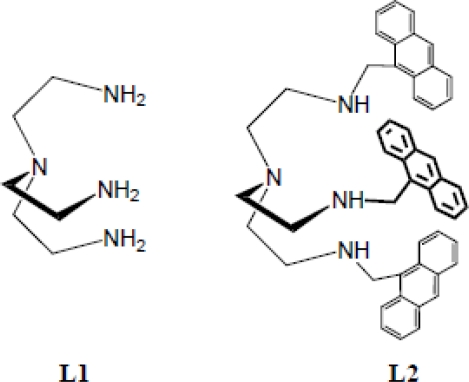
Anion binding hosts: Tren (**L1**) and anthracene-based tren ligand (**L2**).

During the last two decades, the design of synthetic hosts for anion binding has become an important area of research in chemistry [10–25]. Because of the synthetic and structural simplicity, tripodal hosts consisting of three arms received a significant interest in binding a variety of inorganic anions [27]. A tripodal host that is derived from *tris*(2-aminoethyl)amine **L1** (**Scheme 1**) interacts with an anion through electrostatic and hydrogen bonding interactions. For example, simple tripodal amide-based ligands reported by Reinhoudt and coworkers, were shown to bind anions with *C_3_* symmetry [27]. Recently, Ghosh and coworkers reported that pentafluorophenyl-substituted tripodal urea effectively binds phosphate anion through hydrogen bonding interactions from urea units [28,29]. The binding properties are greatly affected by the functional groups attached to tren-based hosts, and some of them showed very high selectivity for anions [26–29]. Increased charges as well as functionalized electron withdrawing groups on ligands are desired in order to achieve high selectivity for an anion. An easy access of a fluorophore to **L1** through primary amines could make this ligand feasible to use as a potential chemosensor for anion sensing. Our continuing interests [21–25] in this area led us to make a simple tripodal chemosensor **L2** that showed significant fluorescence responses to anions with high selectivity for fluoride and sulfate. Herein, we report the synthesis and fluorescence binding studies of **L2,** and the crystal structure of **L1** with sulfate.

## 2. Results and Discussion

### 2.1. Synthesis

The ligand **L2** containing three fluorophore groups, was synthesized by the condensation of **L1** with 9-anthraldehyde and the subsequent reduction of the imine, following the literature method as applied for the related hosts [30]. The neutral ligand **L2** was reacted with 4-toluenesulfonic acid to convert into the corresponding protonated ligand in order to bind an anion. Analysis from the ^1^H NMR spectra suggested that ligand formed an adduct with three tosylates providing three positive charges on the primary amine groups to give a molecular formula, H_3_[**L2**]·(TsO)_3_. The sulfate complex of **L1** was obtained as a microcrystalline solid from the reaction of the ligand with sulfuric acid in methanol. X-ray quality crystals were grown from a slow evaporation of aqueous solution of the salt at room temperature.

### 2.2. Fluorescence Studies

**Figure 1.**
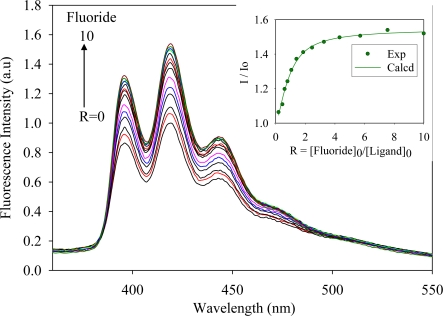
Fluorescence emission spectra of [H_3_**L2**]^3+^ in the presence of (*n*-Bu)_4_N^+^F^−^ in DMSO recorded at the excitation of 310 nm. [Ligand]_0_ = 1.0 × 10^−6^ M. (The inset shows a 1:1 binding isotherm).

**Figure 2.**
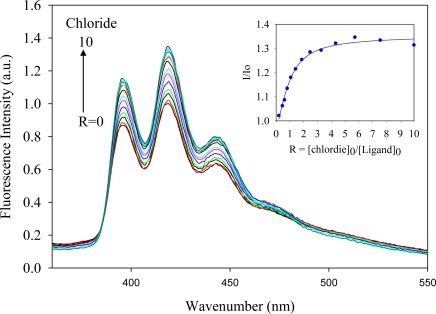
Fluorescence emission spectra of [H_3_**L2**]^3+^ in the presence of (*n*-Bu)_4_N^+^Cl^−^ in DMSO recorded at the excitation of 310 nm. [Ligand]_0_ = 1.0 × 10^−6^ M. (The inset shows a 1:1 binding isotherm).

**Figure 3.**
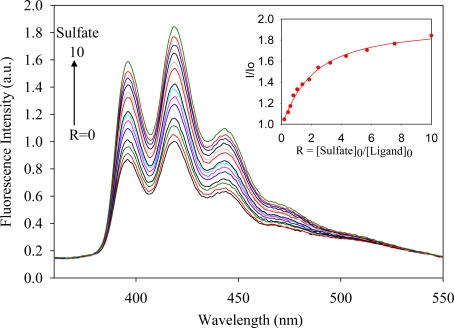
Fluorescence emission spectra of [H_3_**L2**]^3+^ in the presence of (*n*-Bu)_4_N^+^SO_4_^−^in DMSO recorded at the excitation of 310 nm. [Ligand]_0_ = 1.0 × 10^−6^ M. (The inset shows a 1:1 binding isotherm).

**Figure 4.**
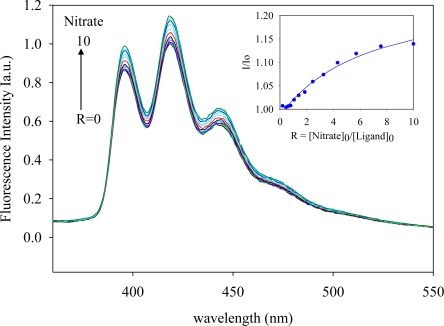
Fluorescence emission spectra of [H_3_**L2**]^3+^ in the presence of (*n*-Bu)_4_N^+^NO_3_^−^ in DMSO recorded at the excitation of 310 nm. [Ligand]_0_ = 1.0 × 10^−6^ M. (The inset shows a 1:1 binding isotherm).

The anthracene-based ligand in its triprotonated form, [H_3_**L2**]·(TsO)_3_, was used in anion binding studies. The ligand contains three tosylates which are known counteranions used in polyamine-based ligands [23–25]. Attempts to determine binding constants using ^1^H NMR titrations were unsuccessful due to negligible chemical shifts of the ligand in the presence of an anion. In the absorption spectra, [H_3_**L2**]^3+^ (1.0 × 10^−4^ M) showed three bands at 354, 371 and 391 nm in DMSO, while the ligand was found to be fluorescence active displaying three bands at 394, 416 and 440 nm when excited at 310 nm, thereby allowing evaluation of its binding properties by fluorescence spectroscopy. The titrations were performed with a variety of anions as *n*-Bu_4_N^+^A^−^ salts (A^−^ = F^−^, Cl^−^, Br^−^, HSO_4_^−^, H_2_PO_4_^−^ and NO_3_^−^) by fluorescence spectroscopy in DMSO at room temperature. Upon the gradual addition of an anion (1 × 10^−4^ M) to the ligand solution, an increase of the fluorescence emission was observed. Figures 1–4 illustrate the representative fluorescence titration spectra derived from the experiments with portionwise additions (0 to 10 equivalents) of the respective anions.

As shown in Figure 1–4, the addition of an anion causes an enhancement of the fluorescence intensity without showing any spectral shift or the formation of an excimer. Such enhancement is attributed to the formation of a ligand-anion complex that restricts the free rotation of the attached fluorophores. A similar observation was previously reported for acyclic benzimidazole-based sensors for halide binding in DMSO [31]. The change in relative fluorescence emission (I/I_0_, where I_0_ and I are the emissions of the ligand before and after the addition of an anion, respectively) as a function of an anion concentration gave the best fit for a 1:1 binding model [32]. The calculated binding data as obtained from non-linear regression analysis are listed in the Table 1. An inspection of these data suggests that the ligand forms strong complexes with halides showing a binding trend of F^−^ > Cl^−^ > Br^−^, which correlates with the electronegativity of the anion. However, the ligand [H_3_**L2**]^3+^ did not show any appreciable spectral change upon the addition of iodide, indicating weak binding. The observed binding constant for fluoride (log *K* = 5.8) is higher than log *K* = 4.93 reported by Lin and coworkers for the same anion with an acyclic benzimidazole-based sensor, as determined by fluorescence titration in DMSO [31]. For the oxoanions, the binding order follows as HSO_4_^−^ > H_2_PO_4_^−^ > NO_3_^−^. The binding constant for nitrate (log *K* = 3.9) observed in the present study is also higher than the corresponding value (log *K* = 3.45) reported by Bianchi and coworkers for the ligand **L1** as determined by potentiometric titration [33]. The overall binding trend is F^−^ > Cl^−^ > Br^−^ > HSO_4_^−^ > H_2_PO_4_^−^ > NO_3_^−^ which does not necessarily support the Hofmeister-like response [34]. The small fluoride with the spherical shape is perhaps more compatible than oxoanions in the tripodal cavity. In this present study, anthracene groups attached to the secondary amines could possibly withdraw electrons from the secondary nitrogens and enhanced the ability to attract an anion by hydrogen bonding and electrostatic interactions, thereby providing an additional stability to the complex.

### 2.3. Crystallographic Studies

Attempts to grow crystals of the ligand **L2** with anions were unsuccessful. Therefore, we proceeded to grow crystals of anions with the precursor **L1** to understand the role of charged hydrogens in binding of an anion, and obtained the X-ray quality crystals of the sulfate of **L1**.

**Figure 5.**
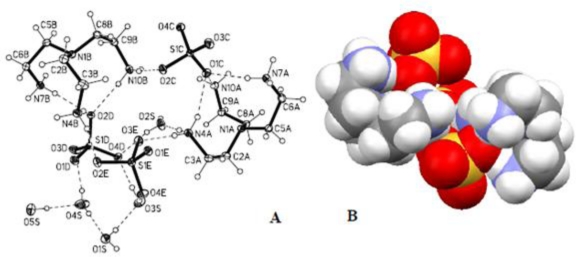
(A) The crystal structure of **[**H_6_(**L1**)_2_]·(SO_4_)_3_·4.5H_2_O showing the atom-numbering scheme and hydrogen **bonding** interactions. Displacement ellipsoids are drawn at the 50% probability level. (B) Space filling model of the sulfate complex showing only three sulfate bonded with two tren units.

**Figure 6.**
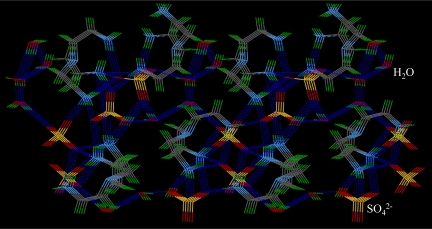
Packing diagram of the sulfate complex of **L1** showing anionic species. Hydrogen bonds are shown in dashed lines.

Structural analysis of the sulfate complex reveals that two tren units were crystallized with three sulfate anions (SO_4_^2−^) to give a molecular formula of 2(C_6_H_21_N)^3+^3(SO_4_)^2−^·4.5(H_2_O). Sulfate complex of the tren was reported before showing five crystalline water molecules [35]. In the complex, each tren is found to be triprotonated with a single charge at each primary nitrogen site. The central amine is not protonated. In the unit cell, two tren units lie on the inversion center of symmetry, facing each other by two arms **(**Figure 5). The third arm slightly moves from the central N-N axis of two trens. A total of six negative charges of the anions are balanced by two triply charged tripodal cations. One water, O5S, was found near a symmetry-related O5S, hence its occupancy was set to 0.5. Thus, a single crystal maintains the same ratio of 1.5 for cation/anion and anion/water molecules. Each of the two tren units adopts a trigonal conformation and holds one sulfate in its pocket by three strong hydrogen bonds (2.73–2.89 Å). The third sulfate species is singly bonded to both hosts through two oxygen atoms and serves as a linker of the two tren units. The central amines of two tren is separated by 8.73 Å that is slightly higher than that observed in the sulfate complex of *m*-xylyl amine cryptand or pyridine amide crptand incorporating same tren units [36]. In packing diagram, both anionic and cationic units are repeated alternatively, forming a layer with extensive hydrogen bonding networks involving water molecules (Figure 6).

## 3. Experimental Section

### 3.1. General

All reagents used in synthesis were reagent grades and were purchased from Aldrich. *n*-Bu_4_N^+^ salts of anions were purchased from TCI America. Nuclear magnetic resonance (NMR) spectra were recorded on a Varian Unity INOVA 500 FT-NMR spectrometer at 500 MHz. Chemical shifts are expressed in ppm and calibrated against TMS or TSP as an external reference in a sealed capillary tube. Absorption spectra were performed by a Varian Cary 3E. Fluorescence experiments were carried out by a spectrofluorometer (Model FluoroMax-2). Crystal structures were determined from the Crystallographic Laboratory at the University of Oklahoma.

### 3.2. Synthesis

**L2:** To a solution of tris-2-aminoethylamine (1 g, 0.00684 mol) in CH_3_OH (30 mL) was added a solution of 9-anthraldehyde (4.23 g, 0.0205 mol) dissolved in CH_3_OH (30 mL). The resulting mixture was stirred at room temperature for 24 hours to give a yellow semi-solid product which was dried under vacuum. The Schiff base product was dissolved in CH_3_OH (100 mL). NaBH_4_ (1.4 g, 0.037 mol) was added to the solution to reduce the imine into the corresponding amine. After stirring at room temperature for 24 hours the solvent was removed in vacuo. The resulting reddish oily residue was dissolved and the aqueous phase was extracted by CH_2_Cl_2_ (3 × 50 mL). The organic layers were combined and dried by adding MgSO_4_ (2 g). The solvent was evaporated under reduced pressure. The residue was purified by column chromatography using neutral alumina (2% methanol in CH_2_Cl_2_) to give a reddish solid. Yield: 70%. ^1^H NMR (500 MHz, CDCl_3_, TMS): *δ* 8.28 (s, 3H, Ar*H*), 8.07 (d, 6H, Ar*H*), 7.88 (d, 6H, Ar*H*), 7.33–7.27 (m, 12H, Ar*H*), 4.40 (s, 6H, ArC*H_2_*), 2.57 (t, 6H, NCH_2_C*H_2_*), 2.42 (t, 6H, NC*H_2_*). Anal. Calcd. for (C_51_H_48_N_4_): C, 85.44; H, 6.75; N, 7.81. Found: C, 85.53; H, 6.72; N, 7.71.

[H_3_**L2**]·(TsO)_3_: The protonated ligand was obtained by reacting 100 mg **L2** with 4-fold *p*-toluenesulfonic acid in methanol. The addition of diethyl ether resulted in a yellowish microcrystalline product that was filtered and washed by diethyl ether. Analysis of the ^1^H NMR spectra suggested that the ligand contained three tosylates groups. Yield: 80%. ^1^H NMR (500 MHz, DMSO, TMS): *δ* 8.83 (s, 3H, Ar(An)*H*), 8.49 (d, 6H, Ar(An)*H*), 8.21 (d, 6H, Ar(An)*H*), 7.59 (m, 12H, Ar(An)*H*), 7.47 (d, 6H, Ar(Ts)*H*), 7.10 (d, 6H, Ar(Ts*H*), 5.31 (s, 6H, ArC*H_2_*), 3.57 (t, 6H, NCH_2_C*H_2_*), 3.07 (t, 6H, NHC*H_2_*), 2.28 (s, 9H, Ar(Ts)C*H_3_*). Anal. Calcd. for (C_69_H_66_N_4_O_6_S_3_): C, 72.48; H, 5.82; N, 4.90. Found: C, 72.68; H, 5.78; N, 4.85.

[H_6_(**L1)**_2_]·(SO_4_)_3_: **L1** (100 mg) was reacted with a few drops of sulfuric acid in methanol. The white precipitate was formed immediately, which was filtered and washed by diethyl ether. Yield: 70%. ^1^H NMR (500 MHz, D_2_O, TSP): *δ* 3.02 (t, 6H, NCH_2_C*H_2_*) 2.76 (t, 6H, NC*H_2_*), ^13^C NMR (125 MHz, D_2_O, TSP): *δ* 49.74 (NCH_2_*C*H_2_), 36.33(N*C*H_2_). This compound was characterized by X-ray analysis.

### 3.3. X-ray Crystallography:

X-ray quality crystals of **L1** with sulfate were grown from a solution of the corresponding salts by slow evaporation at room temperature. The crystallographic results and details of data collection are given in Table 2. Intensity data were collected using a diffractometer with a Bruker APEX CCD area detector [37] and graphite-monochromated *Mo Kα* radiation (λ = 0.71073 Å). The sample was cooled to 100(2) K. Cell parameters were determined from a non-linear least squares fit of 4252 peaks in the range 2.45 < θ < 28.32°. A total of 5688 data were measured in the range 2.45 < θ < 28.32° using ω oscillation frames. The data were corrected for absorption by the semi-empirical method [38] giving minimum and maximum transmission factors of 0.756 and 0.919. The data were merged to form a set of 1368 independent data with R(int) = 0.0291 and a coverage of 100.0%. Cell parameters were determined from a non-linear least squares fit of 6615 peaks in the range 0.00 < θ < 0.00°. The data were merged to form a set of 7427 independent data with R(int) = 0.0312 and a coverage of 100.0%.

Space groups for the complexes were determined by systematic absences and statistical tests and verified by subsequent refinement. The structure was solved by direct methods and refined by full-matrix least-squares methods on *F*^2^ [39]. Hydrogen atom positions bonded to the carbons were initially determined by geometry and refined by a riding model. Hydrogens bonded to nitrogens and oxygens were located on a difference map, and their positions were refined independently. Non-hydrogen atoms were refined with anisotropic displacement parameters. Hydrogen atom displacement parameters were set to 1.2 times the displacement parameters of the bonded atoms. Hydrogen bonding interactions in the crystals are shown in Table 3. A total of 445 parameters were refined against 7427 data to give wR(F2) = 0.0896 and S = 1.010 for weights of w = 1/[σ2 (*F*^2^) + (0.0480 P)^2^ + 0.6000 P], where P = [*F_o_^2^* + *2F_c_^2^*] / 3. The final R(*F*) was 0.0322 for the 6666 observed, [F > 4σ(F)], data. The largest shift/s.u. was 0.001 in the final refinement cycle. The final difference map had maxima and minima of 0.380 and −0.456 e/Å^3^, respectively. In sulfate complex, one water molecule, O5S, was found near symmetry-related O5S, hence its occupancy was set to 0.5.

### 3.4. Fluorescence Studies

Binding properties of [H_3_**L2**]·(TsO)_3_ for the target anions were examined in DMSO by absorption and fluorescence spectrophotometers from the analytical core laboratory at Jackson State University. In the titration experiments, *n*-Bu_4_N^+^A^−^ salts (A^−^ = F^−^, Cl^−^, Br^−^, HSO_4_^−^, H_2_PO_4_^−^ and NO_3_^−^) were used. For the fluorescence measurement, initial concentrations of ligand and anions were 1 × 10^−6^ and 1 × 10^−4^ M, while in the absorption experiment, those values were 1 × 10^−4^ and 1 × 10^−2^ M, respectively. Each titration was performed by 15 measurements at room temperature. The association constant *K* calculated by fitting the change of fluorescence intensity from the non-linear regression analysis of the equations: I/I_0_= ([A]_0_ + [**L**]_0_ + 1/K – (([A]_0_ + [**L**]_0_ + 1/*K*)_2_ – 4[**L**]_0_[A]_0_)^1/2^)ΔI_max_ / 2[**L**]_0_ (where **L** = ligand and A = chloride). Error limit in *K* was less that 20%.

## 4. Conclusions

In this study, we reported the synthesis of an anthracene-based acyclic host and its sensing behavior for two oxoanions, sulfate and nitrate. While the common polyamine based charged receptors are known systems for anions in terms of binding and selectivity, however, their application as anion sensors are rare in literature. During this study, we introduced an anthracene group to a simple tren and made an acyclic sensor showing an excellent response to anions with high association constants in DMSO. The ability of protonated amines to form hydrogen bonds coupled with the electron-withdrawing properties of attached anthracene groups, significantly increased the stability of complexes.

## Figures and Tables

**Table 1.** Association constants (*K*) and free energies (*ΔG*) of the anion complexes of [H_3_**L2**]^3+^ at the excitation of 310 nm at room temperature.

Anion	Log *K^a^*	−Δ*G*/Kcal mol^−1^
Fluoride	5.8	7.9
Chloride	5.5	7.5
Bromide	3.8	5.2
Sulfate	4.8	6.5
Phosphate	4.6	6.3
Nitrate	3.9	5.3

a Association constants determined by fluorescence titrations in DMSO (error limit is less than 20%).

**Table 2.** Crystallographic data for [H_6_(**L1**)_2_].(SO_4_)_3_·4.5H_2_O.

Complex	2(C_6_ H_21_ N)^3+^ 3(SO_4_)^2−^·4.5(H_2_O)
Empirical formula	C_12_ H_51_ N_8_ O_16.5_ S_3_
Formula weight	667.79
Crystal size (mm^3^)	0.50 × 0.21 × 0.11
Crystal system	Triclinic
Space group	*P*1̅
a, Å	8.6555(16)
b, Å	12.247(2)
c, Å	14.919(3)
α, deg	75.836(5)
β, deg	82.716(5)
γ, deg	76.736(6)
Volume (Å^3^)	1,488.3(5)
Z, Z'	2, 1
*d_calc_* g cm^−3^	1.490
λ (Å)	0.71073
T (K)	100(2)
*F*(000)	718
abs coeff (mm^−1^)	0.330
Absorption correction	Semi-empirical from equivalents
Max. and min. transmission	0.967 and 0.850
θ range (°)	1.75 to 28.35
Reflections collected	20,882
Independent reflections	7,427
R(int)	0.0312
*wR*(*F^2^* all data)	*wR2* = 0.0896
*R*(*F* obsd data)	*R*1 = 0.0322
Goodness-of-fit on *F*^2^	1.010
Observed data [I > 2σ(I)]	6,666
Largest and mean shift / s.u.	0.001 and 0.000
Δρ_max ,_ Δρ_min_ (e Å^−3^)	0.380 and −0.456

*wR*2 = {Σ [*w*(*F*_o_^2^ − *F*_c_^2^)^2^] / Σ [*w*(*F*_o_^2^)^2^]}^1/2^, *R*1 = Σ ||*F*_o_| − |*F*_c_|| / Σ |*F*_o_|.

**Table 3.** Selected hydrogen bonding interactions (Å and °) for sulfate complex of **L1**.

D-H...A	d(D-H)	d(H...A)	d(D...A)	<(DHA)
N(4A)-H(4AA)...O(2S)	0.898(18)	1.930(18)	2.8277(17)	179.0(17)
N(4A)-H(4AB)...O(3E)	0.925(18)	1.876(18)	2.7840(16)	166.4(16)
N(4A)-H(4AC)...O(1C)	0.848(19)	2.121(19)	2.8920(16)	151.0(16)
N(4A)-H(4AC)...O(3C)^i^	0.848(19)	2.583(17)	3.0014(16)	111.7(14)
N(4A)-H(4AC)...O(3C) i	0.848(19)	2.583(17)	3.0014(16)	111.7(14)
N(7A)-H(7AA)...O(1E) i	0.898(18)	1.959(18)	2.8559(15)	175.9(16)
N(7A)-H(7AB)...O(1C)	0.874(18)	1.925(19)	2.7957(16)	174.1(16)
N(7A)-H(7AC)...O(2C) i	0.889(18)	1.911(18)	2.7981(16)	175.6(16)
N(10A)-H(10A)...O(1C)	0.847(18)	2.008(18)	2.8238(15)	161.5(16)
N(10A)-H(10B)...O(1E) ii	0.906(18)	1.926(18)	2.7961(16)	160.4(15)
N(10A)-H(10C)...O(4C) iii	0.896(18)	1.904(19)	2.7862(15)	167.9(16)
N(4B)-H(4BA)...O(1D) ^iv^	0.914(19)	1.838(19)	2.7463(16)	172.2(16)
N(4B)-H(4BB)...O(2D)	0.878(19)	2.022(19)	2.8838(17)	166.9(16)
N(4B)-H(4BC)...O(2E)	0.882(19)	2.056(19)	2.8821(16)	155.5(16)
N(4B)-H(4BC)...O(3E)	0.882(19)	2.330(18)	2.9970(17)	132.5(15)
N(7B)-H(7BA)...O(2D)	0.860(18)	2.029(19)	2.8748(16)	167.5(16)
N(7B)-H(7BB)...O(3D) v	0.911(18)	1.911(19)	2.8006(16)	165.0(16)
N(7B)-H(7BC)...O(2E) ^iv^	0.886(18)	1.891(19)	2.7748(15)	175.1(16)
N(10B)-H(10D)...O(2C)	0.898(18)	1.964(19)	2.8569(16)	172.3(16)
N(10B)-H(10E)...O(2D)	0.872(18)	1.981(19)	2.8494(15)	174.0(16)
N(10B)-H(10F)...O(1E) ii	0.899(18)	1.981(18)	2.8686(15)	169.0(16)
N(10B)-H(10F)...O(2E) ii	0.899(18)	2.612(17)	3.1698(16)	121.0(13)

Symmetry transformations used to generate equivalent atoms:

i −x+1, −y, −z+1,

ii x−1, y, z,

iii −x, −y, −z+1,

v −x+1, −y+1, −z,

v −x, −y+1, −z,

vi x, y+1, z.
